# Utility of the Morgan Fingerprint in Structure-Based Virtual Ligand Screening

**DOI:** 10.1021/acs.jpcb.4c01875

**Published:** 2024-05-24

**Authors:** Hongyi Zhou, Jeffrey Skolnick

**Affiliations:** Center for the Study of Systems Biology, School of Biological Sciences, Georgia Institute of Technology, Atlanta, Georgia 30332, United States

## Abstract

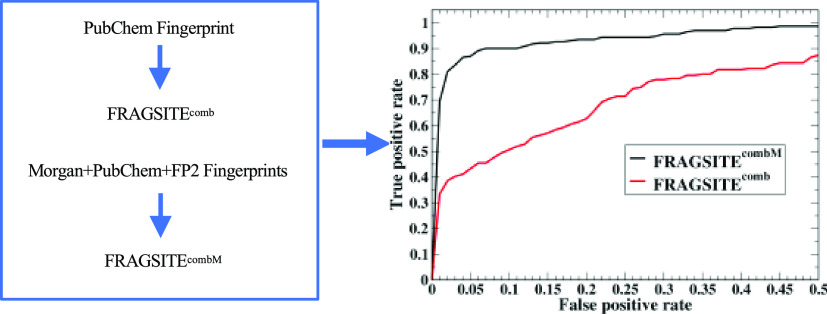

In modern drug discovery, virtual ligand screening (VLS) is frequently applied to identify possible hits before experimental testing and refinement due to its cost-effective nature for large compound libraries. For decades, efforts have been devoted to developing VLS methods with high accuracy. These include the state-of-the-art FINDSITE suite of approaches FINDSITE^comb2.0^, FRAGSITE, and FRAGSITE2 and the meta version FRAGSITE^comb^ that were developed in our lab. These methods combine ligand homology modeling (LHM), traditional ligand similarity methods, and more recently machine learning approaches to rank ligands and have proven to be superior to most recent deep learning and large language model-based approaches. Here, we describe further improvements to our previous best methods by combining the Morgan fingerprint (MF) with the originally used PubChem fingerprint and FP2 fingerprint. We then benchmarked FINDSITE^comb2.0M^, FRAGSITE^M^, FRAGSITE2^M^, and the composite meta-approach FRAGSITE^combM^. On the 102 target DUD-E set, the 1% enrichment factor (EF_1%_) and area under the precision-recall curve (AUPR) of FRAGSITE^comb^ increased from 42.0/0.59 to 47.6/0.72. This 0.72 AUPR is significantly better than that of the state-of-the-art deep learning-based method DenseFS’s AUPR of 0.443. An independent test on the 81 targets DEKOIS2.0 set shows that EF_1%_/AUPR increases from 18.3/0.520 to 23.1/0.683. An ablation investigation shows that the MF contributes to most of the improvement of all four approaches. Thus, the MF is a useful addition to structure-based VLS.

## Introduction

In modern drug discovery, virtual ligand screening (VLS) uses computational tools to discover small molecules that might bind a protein target. In practice, there are two broad categories of traditional VLS methods: (a) structure-based docking methods that use the high-resolution, three-dimensional (3D) structure of a protein target, and dock ligand structures to the target protein;^1−4^ they then evaluate and rank the docked ligand structures using physics, machine learning, or knowledge-based scoring functions.^3,5−7^ The advantage of docking methods is that they have the potential to discover novel binders to the target, whereas their disadvantage is that they are computationally expensive, require high-resolution protein structures, and most importantly, are less accurate than alternatives; (b) ligand-based methods use known ligands that bind to a given protein target to predict new binders based on their similarity to the physical-chemical properties of the known ligands.^8−11^ The advantage of ligand-based methods is that they are usually more accurate than docking, but they cannot discover novel small molecule binders that are chemically quite distinct from known binders and are not able to screen proteins lacking known binders. The need for high-resolution structures and known binders was addressed in our laboratory by ligand homology modeling (LHM)-based methods. Our FINDSITE suite of methods conceptually has the advantages of both traditional methods.^12−16^ They use lower-resolution as well as high-resolution structures of protein targets to find similar binding pockets (template pockets) in the protein–ligand complex structures found in the Protein Data Bank (PDB);^17^ such pockets need not come from evolutionarily related proteins. They then use the corresponding ligands bound in the pockets of PDB structures as template ligands. To further expand the set of such template ligands, two additional databases ChEMBL^18^ and DrugBank^19^ for domain structure comparison^13,14^ were employed. These template ligands are then used in a similar manner as ligand-based methods. Thus, the FINDSITE suite has the advantage of having an accuracy comparable to ligand-based methods for those proteins having known binders, but importantly, they can be applied to proteins without known binders. These methods are also computationally much less expensive than docking methods that not only require a high-resolution target structure but also require the 3D structures of the screened compounds. Our latest work FRAGSITE2 further addressed the issue of LHM methods’ disadvantage in discovering novel binders by decoupling the template ligand information from the machine learning scoring function.^20^ Thus, FRAGSITE2 can be considered as a structure-based method without a similarity search step. FRAGSITE2 was benchmarked on the DUD-E set^7^ and outperforms the latest deep learning-based DenseFS scoring function for VLS.^21−23^ Our protein pocket-based features were compared to the deep learning pretrained large language model (LLM) of protein features used in ConPLex,^23^ and our protein pocket-based features show better performance.

Although FRAGSITE2,^20^ as well as its ancestor FRAGSITE,^20^ outperforms the state-of-the-art deep learning-based scoring function plus docking approaches, e.g., DenseFS^22^ in terms of ROC enrichment (ROCEF_1%_: 49.11 vs 48.0) and area under precision-recall curve (AUPR: 0.465 vs 0.443), there is still additional room for improvement as a perfect ROCEF_1%_/AUPR should be 100/1.0. We previously explored using protein language models for feature selection and found them inferior to our pocket-based features.^20^ In this work, we improve upon FINDSITE^comb2.0^, FRAGSITE, and FRAGSITE2 by exploring a new representation of the ligand that not only includes the previously used FP2^24^ fingerprint in FINDSITE^comb2.0^ and the PubChem fingerprint^25^ in FRAGSITE and FRAGSITE2, but we now include the Morgan fingerprints (MF) employed in the ConPLex^23^ work.^26^ Unlike other fingerprints, the MF employs an extended-connectivity fingerprint ECFP4,^27^ and thus, it has knowledge of the connectivity of the functional groups in the ligand. In contrast, PubChem is a fragment/substructure-based fingerprint approach without connectivity information other than atom bond types. The FP2 fingerprint is a path-based fingerprint which indexes small molecule fragments based on linear segments of up to 7 atoms. FP2 indexes only linear fragments (ignoring rings and single atoms), while PubChem considers various substructures (e.g., an element count, type of ring system, atom pairing, atom environment of nearest neighbors, etc., in a chemical structure). Thus, these fingerprints represent different small molecule properties. ECFP4 (of which the MF is an extension) was shown to perform the best in similarity-based approaches.^28^ Therefore, an improvement in both FINDSITE^comb2.0^ and FRAGSITE that have a similar searching component is expected. In practice, we will update these methods using the strategy described here. As shown below, the use of the MF results in a significant improvement in all variants of our VLS algorithms. Thus, the MF is not only useful for similarity-based approaches but also for developing a scoring function for structure-based methods. In what follows, we shall denote the MF augmented approach X as X^M^, e.g., FRAGSITE2 as FRAGSITE2^M^.

## Materials and Methods

**Figure 1:**
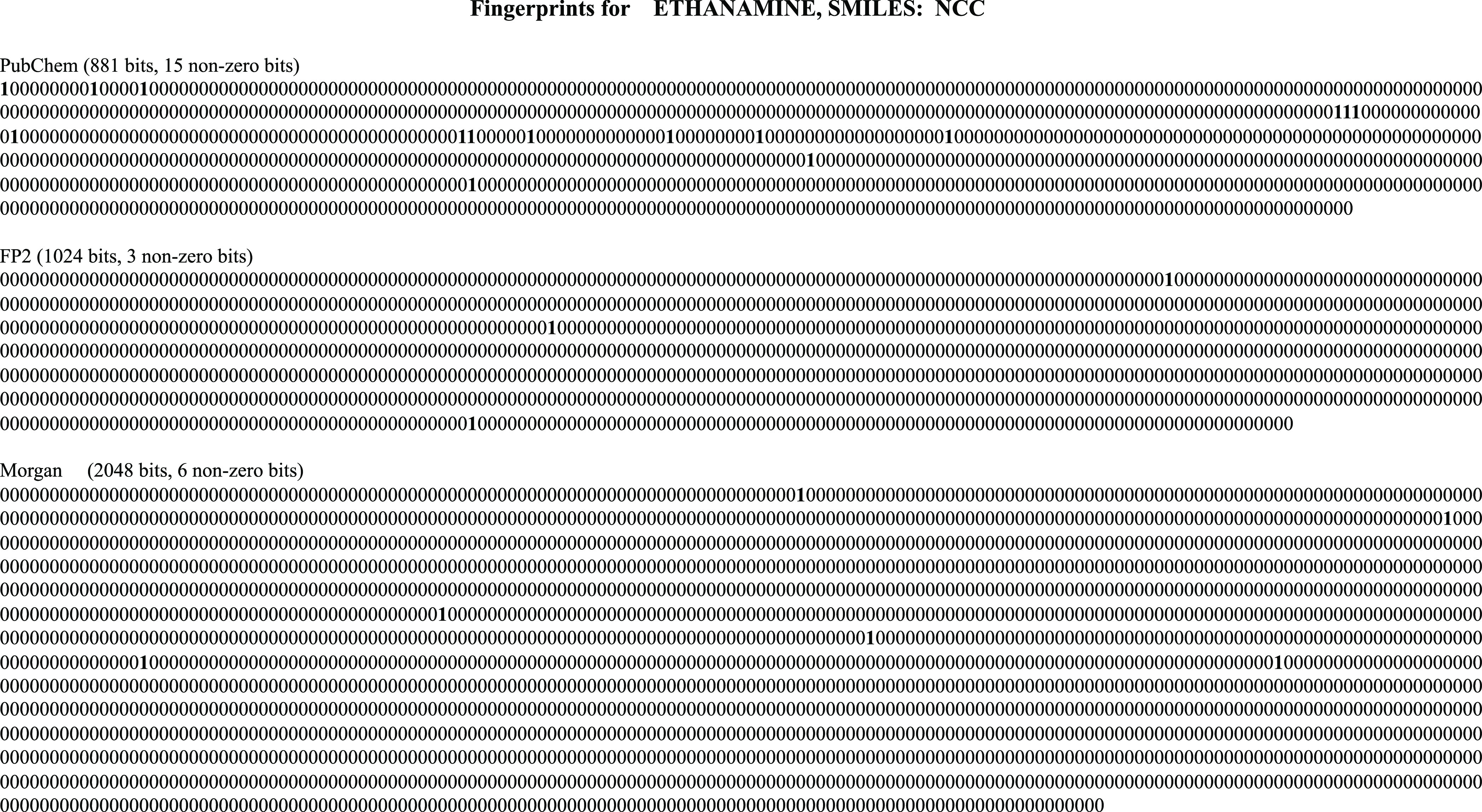
Example of PubChem, FP2, and Morgan fingerprints for ethanamine.

We first describe how the various ligand fingerprints are implemented. We derive 20-dimensional feature vectors from the template pockets using the mean amino acid composition of all template pockets. Then, this feature vector, for a given target-ligand pair, is concatenated with the 3953 dimension fingerprints of the PubChem fingerprint^25^ computed by the PaDEL-descriptor.^29^ FP2 fingerprints are calculated using Open Babel,^30^ and the Morgan fingerprint (MF) is calculated using the python package RDKit with a radius = 2, fpSize = 2048 setting (RDKit: Open-source cheminformatics; http://www.rdkit.org). To have an idea what these fingerprints look like, Figure 1 shows an example of PubChem, FP2, and Morgan fingerprints for ethanamine. PubChem is a substructure-based fingerprint that defines 881 substructures. If one substructure is present, the corresponding bit is set to 1, otherwise 0. FP2 indexes small molecule fragments based on linear segments of up to 7 atoms and MF indexes atom types and their connectivities. The indexes are represented by hash values, and then, hash values are divided by 1024 for FP2 and 2048 for MF. The bit position corresponding to the remainder (modulus) plus one is set to 1. Thus, the set bits of FP2 and MF are just indexes. Due to the much larger dimension of the feature vector of the MF, compared to FRAGSITE2 (3,973 vs 901), rather than use boosted tree regression, we employed a similar but much faster Extreme Gradient Boosting (XGB) regression machine learning method.^31^ XGB is optimized for memory usage and computational efficiency (e.g., for sparse matrix, parallel tree computing), has similar performance to boosted trees, and is less likely cause overfitting. In practice, the GradientBoostingRegressor implemented in the Scikit-learn kit^32^ was applied with the following parameters: n_estimators = 3000, max_depth = 6, and learning_rate = 0.05. With these parameters, we slightly improved upon our original FRAGSITE2 result with EF_1%_/AUPR of 33.14/0.468 compared to 32.72/0.465 of our boosted tree result with 1500 steps. On using XGB with 1500 estimators, we obtain an EF_1%_/AUPR of 32.37/0.450 indicating that XGB requires more trees to achieve the same performance as our boosted tree approach.

**Figure 2:**
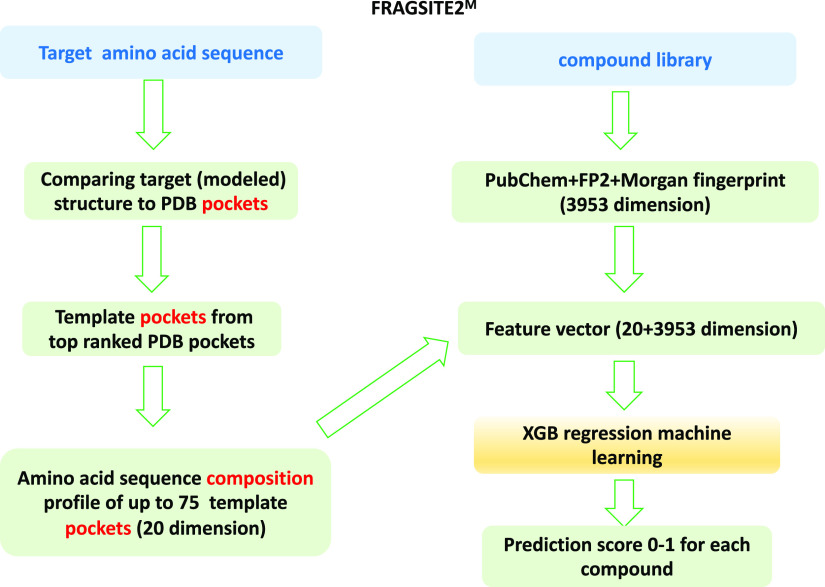
FRAGSITE2^M^ flowchart.

FRAGSITE2^M^’s flowchart is shown in Figure 2; in practice, the approach is very similar to FRAGSITE2 with a few updates.^20^ FRAGSITE2^M^ employs the FINDSITE^filt^ component of FINDSITE^comb2.0^^13^ that only uses a PDB protein–ligand complex for binding site prediction and for deriving template ligand fingerprints. However, the template ligands themselves are not used in FRAGSITE2^M^ for searching for possible new binders but only for determining the binding pocket of the target.

**Figure 3:**
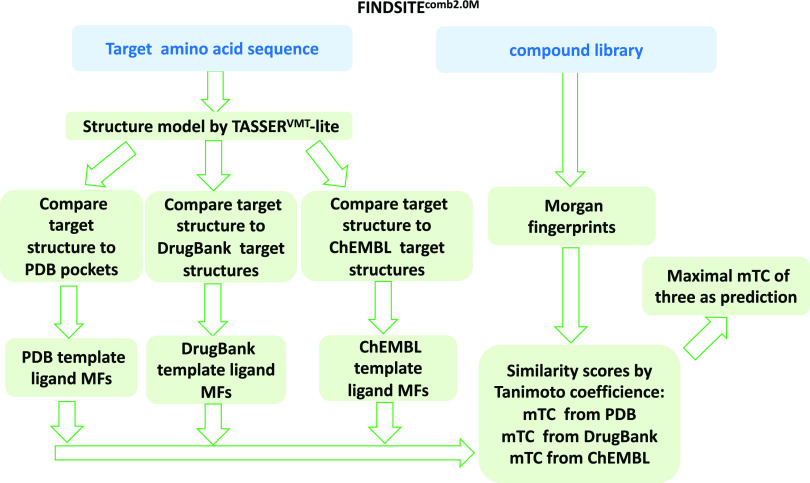
FINDSITE^comb2.0M^ flowchart.

In FINDSITE^comb2.0^, a template protein must have a TM-score^33^ > 0.6 to the target protein’s structure and at least 80% of the template sequence must be aligned to the target sequence. A sequence cutoff is applied in benchmarking mode to exclude templates whose sequence identity > the cutoff for selecting template pockets. Then, template pockets are selected using up to the top 75 pockets from the PDB ligand-protein complex structures.^13^ This part of FRAGSITE2^M^ is the same as in FRAGSITE2.^20^ The procedure used for FINDSITE^comb2.0M^ (shown Figure 3) is exactly the same as FINDSITE^comb2.0^,^13^ but we replace the original FP2 fingerprint with the MF for the ligand representation.^13^ Interestingly, we found that using the combined FP2, PubChem, and MF fingerprints does not give better performance than MF alone. This issue will be further addressed in the DISCUSSION section.

**Figure 4:**
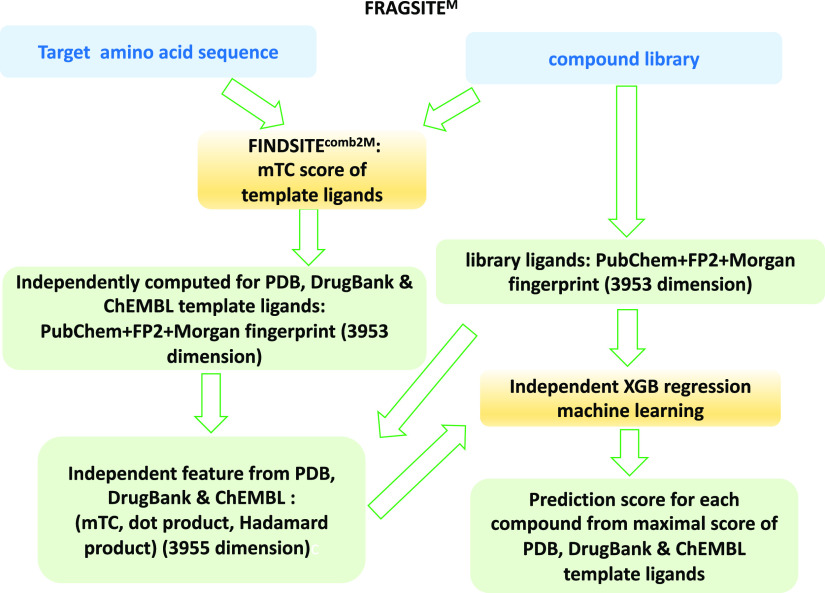
FRAGSITE^M^ flowchart.

In FRAGSITE^M^ (Figure 4), the original 881 bits PubChem fingerprint was replaced with combined FP2, PubChem, and MF fingerprints similar to FRAGSITE2^M^. Again, due to the larger number of features, the original boosted tree regression has been updated with XGB regression with double the number of trees of original FRAGSITE and uses the following parameters: n_estimators = 300, max_depth = 6, and learning_rate = 0.05.

**Figure 5:**
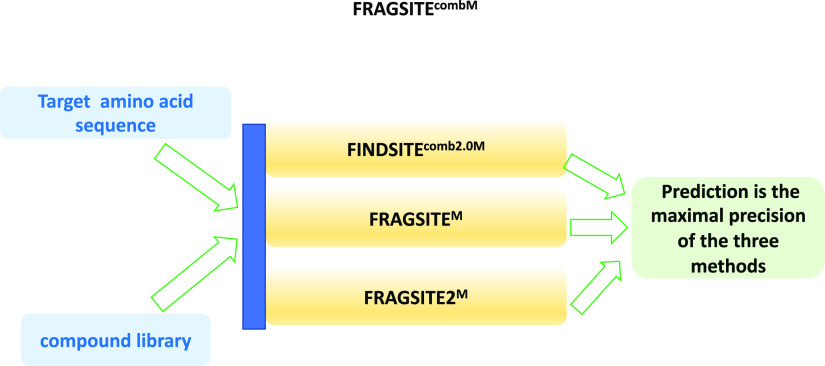
FRAGSITE^combM^ flowchart.

The meta-approach FRAGSITE^combM^ (Figure 5) simply takes the best predicted precision of each screened molecule as the prediction. Finally, we convert the mTC score from FINDSITE^comb2.0M^ and the machine learning scores from FRAGSITE^M^ and FRAGSITE2^M^ to the predicted precision according to eq 2 below.

### Training and Testing Data Sets

Similar to FRAGSITE2, we used the DUD-E^34^ ligand virtual screening benchmark data set for both training and testing. A modified leave-one-out cross-validation (LOOCV) was carried out by excluding all targets having a sequence identity >80% to the given tested protein target. We used the DUD-E^34^ ligand virtual screening benchmark data set for both training and testing. The 80% identity cutoff is used only for template pocket selection and training target inclusion for a given testing target for fair comparison to other state-of-the-art methods such as the DenseFS scoring function^22^ and RF-score-VS^7^ that used this cutoff to separate training and testing targets. However, since we are using predicted target structures of the target, while DenseFS and RF-score-VS used crystal structures, for structure modeling, we still applied a 30% sequence cutoff to select templates that were used to predict the structure of target protein.^35^ In training using XGB regression for ligand-protein binding, the objective function value is assigned as 1 if the molecule is a true binder of the target (in the DUD-E benchmarking set,^34^ the active ligands) and 0 if the molecule is not a binder (decoys in DUD-E). Since overall DUD-E has an active to decoy ratio around 0.016, we randomly picked ∼10% decoys and used all actives in training, resulting in around 160,000 protein–ligand pairs which is a slightly balanced positive/negative ratio.

To avoid any bias in the DUD-E set that favors a machine learning method with training and testing on the same set, we also tested FRAGSITE2^M^ on an independent set from the training DUD-E set, DEKOIS2.0.^36^ This set has 81 structurally diverse targets with an active to decoy ratio around 0.033 and is based on BindDB bioactivity data.^37^ Compared to previous work, we again use an 80% instead of 30% sequence identity cutoff between testing targets and training DUD-E targets. Again, a 30% sequence cutoff to templates is applied for structure modeling of targets.^35^ We note that there are some other VLS benchmarking sets, e.g., MUV^38^ and LIT-PCBA.^39^ However, since the MUV set has a total of only 17 targets and LIT-PCBA has 15 targets, they represent a very small number of protein families, and the small number of targets make them statistically insufficient to distinguish between methods. The performance of one or a few outliers could dominate the overall performance. Thus, they were not considered.

### Assessment

In modern drug discovery, the screened compound library could be immense, for example, Stein et al. docked 150 million molecules to an MT1 crystal structure;^40^ 1% or even 0.01% of molecules are still too many for experimental testing. Thus, instead of using the area under receiver operating characteristic curve (AUROC), we use the more meaningful, interpretable enrichment factor at the top *x* fraction (or 100*x*%) of the ranked list defined as
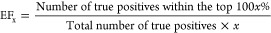
1

To compare the DenseFS score,^22^ for a cutoff independent evaluation, we prefer AUPR, the area under the precision-recall curve^41^ to the AUC (area under the ROC curve), and the ROC 1% enrichment factor (ROCEF_1%_: enrichment at a 1% false-positive rate) that is slightly different from the above EF_1%_. Although ROCEF_1%_ eliminates the database dependence (e.g., the maximal possible value for ROCEF_1%_ is always 100 as opposed to EF_1%_ that depends on the active/negative ratio of the data set), it is not easily related to the design of experimental validation (e.g., the cutoff of 1% false positives is not easy to perform in practice; sometimes it could give you a long list, other times a shorter one). AUPR is a better measure than AUC to distinguish the ability of methods to rank positives in the very top ranks when true positives are rare and only the very top ranked ones are tested as is often the case in VLS.^41^

In practice, as was previously done with FINDSITE^comb2.0^, FRAGSITE, and FRAGSITE2, we also report the predicted precision for a given machine learning score *S*_frg_:
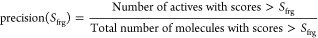
2

The precision score is useful for judging if the prediction is confident or not. To derive the predicted precision, we merge all the LOOCV predictions for actives and decoys of all targets from the DUD-E data set.^34^ This precision score is used to combine the FINDSITE^comb2.0M^, FRAGSITE^M^, and FRAGSITE2^M^ methods to form the meta-method FRAGSITE^combM^ by using the best predicted precision of these three methods for a given molecule.

## Results

### Benchmarking on the DUD-E Set

The DUD-E set^34^ is commonly used by VLS methods for benchmarking and training of machine learning scoring functions.^21^ Although some of the machine learning scoring functions have been trained on pairwise protein–ligand binding affinity or classification,^21^ they are usually not good for VLS as demonstrated by the RF-score-VS study.^7^ Importantly, classification predictions are not suitable for rank ordering of molecules. In practice, they cannot be applied for screening large compound libraries because the positive classes still need a ranking list and classification predictions will give a random order among predicted positives. FRAGSITE2^M^ and its component algorithms also use the DUD-E set for training and cross-validation testing. Here, cross-validation is performed by removing proteins having an amino sequence identity >80% to the given testing target from training. This is similar to the so-called “vertical split” scenario of the RF-score-VS.^7^

Table 1 summarizes FRAGSITE2^M^’s results in comparison to original FINDSITE^comb2.0^, FRAGSITE, FRAGSITE2, FRAGSITE^comb^ and updated FINDSITE^comb2.0M^, FRAGSITE^M^, and FRAGSITE^combM^. To test which of the three fingerprints (FP2, PubChem, MF) are most important, we carried an ablation study and denoted them as FRAGSITE2^M^-noFP2, FRAGSITE2^M^-noPubChem, and FRAGSITE2^M^-noMF, respectively. For comparison to the latest literature, we also included DenseFS^22^ that used AutoDock Vina for docking poses^2^ and family-specific training of a deep convolution neural network (CNN) scoring function.

**Table 1:** Mean Enrichment Factor EF_1%_ and AUPR of Different Methods on the 102 Target DUD-E Set^a^

Method^a^	EF_1%_	AUPR
DenseFS^22^		0.443
**Original Versions**		
FINDSITE^comb2.0^	37.2	0.508
FRAGSITE	41.5	0.591
FRAGSITE2^20^	32.7	0.465
FRAGSITE^comb^	41.7	0.606
updated versions		
FINDSITE^comb2.0M^	42.0	0.591
FRAGSITE^M^	45.4	0.662
FRAGSITE2^M^	38.0	0.557
FRAGSITE^combM^	**47.6**	**0.712**
**Ablation Versions**		
FRAGSITE2^M^-noFP2	37.5	0.546
FRAGSITE2^M^-noPubChem	37.2	0.542
FRAGSITE2^M^-noMF	35.9	0.516
FRAGSITE2^M^-onlyMF	35.4	0.514

a Boldface numbers represent the best performing results.

**Figure 6:**
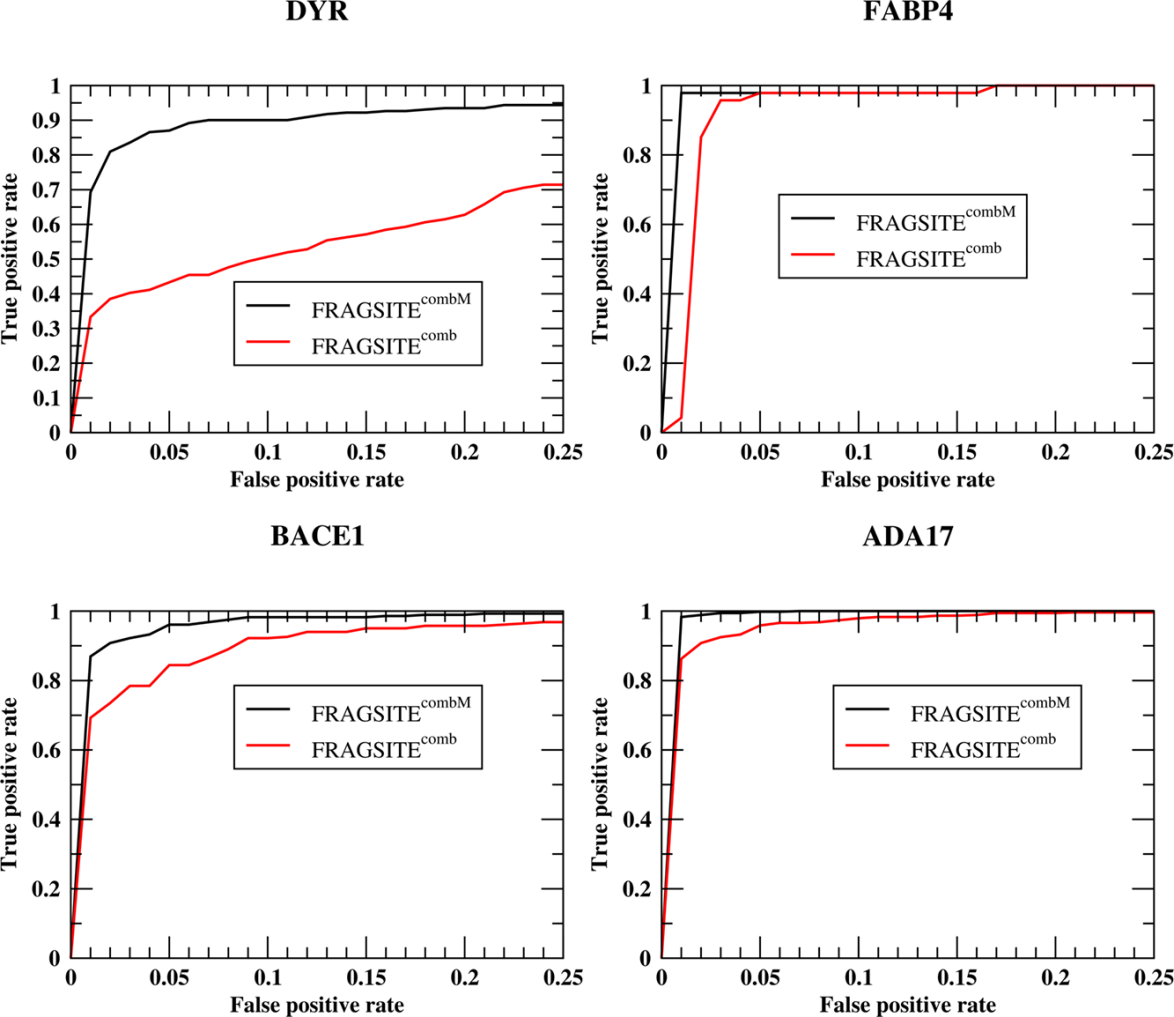
ROC curves of the best performing FRAGSITE^combM^ for 4 example DUD-E targets: DYR, FABP4, BACE1, and ADA17.

In Table 1, we note that the DUD-E set has an average decoy/active ratio of around 60 resulting in a maximal average EF_1%_ = 61.08 for the case of perfect ranking. Compared to the state-of-the-art deep learning-based method DenseFS scoring function^22^ using the AUPR as the relevant metric (0.557 vs 0.443), FRAGSITE2^M^ is better by 26%. Compared to original FRAGSITE2 that uses only PubChem type substructures for ligand features, FRAGSITE2^M^ has a +16.0% (+20%) increase for EF_1%_(AUPR) (38.00/0.557 vs 32.7/0.465). Ablation results in Table 1 show that removing the MF fingerprint has the largest reduction: EF_1%_/AUPR drops down to 35.90/0.516 or a −5.5%/-7.4% reduction, whereas removing FP2 and PubChem leads to 37.54/0.546 and 37.15/0.542 with PubChem reduced slightly more than FP2. If we only use the MF (FRAGSITE2^M^-onlyMF), the performance (EF_1%_/AUPR = 35.42/0.514) is very close to using the combined FP2 and PubChem (noMF:35.90/0.516). Thus, as a structure-based approach, FRAGSITE2^M^ significantly outperforms the DenseFS scoring function and FRAGSITE2. The improvement over FRAGSITE2 is around half from the Morgan fingerprint and half from the combined FP2 and PubChem fingerprints. Inevitably, the similarity-based approaches FINDSITE^comb2.0M^ and FRAGSITE^M^ with EF_1%_/AUPR of 42.0/0.591 and 45.4/0.662 are still better than the structure-based FRAGSITE2^M^ as they tend to perform best in the regime of significant target/template ligand similarity. The meta-approach FRAGSITE^combM^ is better than any individual component method with EF_1%_/AUPR of 47.6/0.712. Thus, the predicted EF_1%_ is 78% of the maximal average enrichment factor; that is, it recovers 78% of all possible ligands. Moreover, the AUPR increases by 60% from that provided by DenseFS.^22^ The AUPR improvement of current methods over previous methods indicates that the improvements are from better top ranked true positives. To further demonstrate that this is true, in Figure 6, we show the ROC curves of the best performing FRAGSITE^combM^ for 4 example targets: DYR (AUPR from 0.26 to 0.63), FABP4 (0.35 to 0.85), BACE1 (0.62 to 0.88), and ADA17 (0.80 to 0.94). Indeed, the improvements at around 1% false-positive rate are larger than those of higher false-positive rates.

### Testing on the DEKOIS2.0 Data Set

The DEKOIS2.0 data set^36^ was used to benchmark FRAGSITE2^M^ and its components as an independent testing data set from the training DUD-E set. This was done to show if the improvement is generalizable to independent sets or has somehow just been overtrained. DEKOIS2.0 is based on BindDB bioactivity data^37^ and provides 81 structurally diverse benchmark sets for a wide variety of different target classes. It has a decoy/active ratio of 30; meaning the maximal possible EF_1%_ is 30, which is smaller than the ∼60 found for the DUD-E set. To enable direct comparison to other previous methods, we again use an 80% sequence identity cutoff between testing the DEKOIS2.0 targets and the training DUD-E targets. Table 2 summarizes the performance on the DEKOIS2.0 set of FRAGSITE2^M^; its individual components FRAGSITE2, FINDSITE^comb2.0M^, and FRAGSITE2^M^; and the results from the RF-score-VS scoring function.^7^ Consistent with the DUE-E set results, the EF_1%_/AUPR of FRAGSITE2^M^ increases from 15.9/0.437 to 18.5/0.551. Compared to previous work of RF-score-VS that employed a random forest machine learning with structure features derived from docked poses of the docking method, the EF_1% of_ FRAGSITE2^M^ is almost twice as large as its EF_1%_ = 9.84. Again, the similarity-based FINDSITE^comb2.0M^ and FRAGSITE2^M^ are still better with EF_1%_/AUPR of 20.8/0.565 and 20.1/0.589, respectively. The meta-method FRAGSITE^combM^ is the best with EF_1%_/AUPR = 23.1/0.683. Once again, EF_1%_ has significantly improved and is 77% of the maximal possible value; interestingly, this is essentially the same recall rate as in the DUD-E set.

**Table 2:** Mean Enrichment Factor EF_1%_ and AUPR of Methods on the 81 Target DEKOIS2.0 Set^a^

method^a^	EF_1%_	AUPR
RF-score-VS^7^	9.84	
FRAGSITE2^20^	15.9	0.437
FINDSITE^comb2.0M^	20.8	0.565
FRAGSITE^M^	20.1	0.593
FRAGSITE2^M^	18.5	0.551
FRAGSITE^combM^	**23.1**	**0.683**

a Boldface numbers represent the best performing results.

## Discussion

Our results show that by combining the fingerprints computed just from a small molecule’s one-dimensional (1D) SMILES string that are used in conventional machine learning (as opposed to deep neural networks), for the DUE-E set, the EF_1%_/AUPR of FRAGSITE2 can be improved from 32.7/0.465 to 38.0/0.557. Although FRAGSITE2^M^ is not a traditional structure-based VLS method that needs high-resolution 3-dimensional (3D) structures of both the protein target and binding ligands, it outperforms those traditional methods that were coupled with deep machine learning/RF scoring functions such as DenseFS and RF-score-VS. In traditional methods with machine learning approaches, features are usually derived from docked 3D structure poses, such features include knowledge-based contact energy, empirical physical interaction energy, etc. The errors in docked poses and energy functions will limit the accuracy of machine learning. FRAGSITE2^M^, on the other hand, only uses properties from the template pockets that are experimentally determined and from the ligand-side its pocket sequence. Machine learning will extract which of the features from the ligand’s intrinsic structure are likely to pair with which amino acids in the pocket. Better representation of both target features and ligand features are the key to our better performance. Our previous work, FRAGSITE2, shows that using the deep learning pretrained LLM of protein for target feature (1024 component) will diminish its performance which indicates that template pocket amino acid composition (20 component) is a better feature to employ. Our current work shows that 2048 bits of the Morgan fingerprint are more predictive than the original 881 bits of PubChem and the 1024 bits of FP2 fingerprints. However, the latter two still have a complementary effect to the MF in that combining them gives better performance than just the MF alone. However, we also find that, for the similarity-based method FINDSITE^comb2.0M^, the combined fingerprints do not give better performance than just use of the MF. This is due to the fact that the similarity scores in FINDSITE^comb2.0M^ have equal weights for every fingerprint bit. Thus, the less accurate ones from FP2 and PubChem will drag down the overall performance; this is unlike the machine learning scores in FRAGSITE^M^ and FRAGSITE2^M^ that learn from the useful bits and ignore the less informative ones.

By combining FRAGSITE2^M^ with the similarity-based methods FINDSITE^comb2.0M^ and FRAGSITE^M^ using the derived precision scores, the meta-approach FRAGSITE^combM^, which is better than individual methods, has an EF_1%_/AUPR 47.6/0.72. Similarly, for the DEKOIS2.0 sets, FRAGSITE^combM^ has an EF_1%_/AUPR = 23.1/0.683 Thus, its recall is close to 80% of actives/positives within the top 1% of the screened library for both the DUE-E and DEKOIS2.0 sets. These significant improvements are around half due to the Morgan fingerprint.

Why is the MF better than FP2 or PubChem? It could be that FP2 and PubChem consider fragments/substructures as independent collections; thus, randomly permuting the fragments results in no change in their values, while the connectivity information in the MF will sense this change. Although we have explored the LLM for targets, we still have the option of using a relatively larger sequence-trained LLM or perhaps develop a pocket-based LLM for further improvement of FRAGSITE2^M^. Furthermore, we shall also explore the possibility of developing a LLM for ligand or even further pocket-ligand pairs. Another possibility similar to the ligand’s MF is to consider the connectivity of the template pockets rather than just treating it as a collection of amino acids without a given order. Given the present encouraging results, these issues will be explored in future work.
